# Pericervical Dentin Preservation: Comparative Study of Three Reciprocating Systems

**DOI:** 10.21142/2523-2754-1303-2025-250

**Published:** 2025-08-31

**Authors:** Daniel López-Velasco, Carmen Rojano-Buelvas, Liceth Rodríguez-Wilches, Javier Alvear-Pérez, José Flórez-Ariza, Luisa Barriga-Periñán, Jaime Plazas-Román

**Affiliations:** 1 DDS, Specialist in Endodontics, Universidad de Cartagena. Cartagena, Colombia. dlopezv4@unicartagena.edu.co, crojanob@unicartagena.edu.co, lrodriguezw@unicartagena.edu.co, Universidad de Cartagena Universidad de Cartagena Cartagena Colombia dlopezv4@unicartagena.edu.co crojanob@unicartagena.edu.co lrodriguezw@unicartagena.edu.co; 2 DDS, Specialist in Endodontics, Professor at Universidad de Cartagena. Cartagena, Colombia. jalvearp@unicartagena.edu.co Universidad de Cartagena Universidad de Cartagena Cartagena Colombia jalvearp@unicartagena.edu.co; 3 DDS, MSc in Oral and Maxillofacial Radiology, Professor at Universidad de Cartagena. Cartagena, Colombia. jfloreza3@unicartagena.edu.co Universidad de Cartagena Universidad de Cartagena Cartagena Colombia jfloreza3@unicartagena.edu.co; 4 DDS, Master in Epidemiology with emphasis on IR and Social Health Policy, Professor at Universidad de Cartagena. Cartagena, Colombia. lbarrigap@unicartagena.edu.co Universidad de Cartagena Universidad de Cartagena Cartagena Colombia lbarrigap@unicartagena.edu.co; 5 DDS, MSc in Bioinformatics, Professor at Universidad de Cartagena; Professor at Universidad del Sinú, Cartagena campus. Cartagena, Colombia. jplazasr@unicartagena.edu.co Universidad del Sinú Universidad del Sinú Cartagena Colombia jplazasr@unicartagena.edu.co

**Keywords:** endodontics, dentin, root canal preparation, cone-beam computed tomography, endodoncia, dentina, preparación del conducto radicular, tomografía computarizada de haz cónico

## Abstract

**Introduction:**

Endodontic treatment aims to disinfect and shape root canals through controlled wear of their walls. Selecting the appropriate rotary system is essential for preserving pericervical dentin. To compare pericervical dentin wear produced by three reciprocating

**Objective:**

systems (WaveOne Gold, One Reci, and Reciproc Blue).

**Methodology:**

An in vitro experimental study was conducted using 30 single-rooted premolars, randomly distributed into three groups of 10 teeth each. Tomographic evaluations were performed before and after instrumentation, measuring mesial, distal, palatal/lingual, and buccal surfaces at 4 mm from the cemento-enamel junction. The Kruskal-Wallis test was used to compare the three systems, with Dunn's post-hoc test for pairwise comparisons (significance level p<0.05).

**Results:**

A statistically significant difference was found in the distal sector (p=0.005), with One Reci causing the least wear (0.05±0.02mm) compared to WaveOne Gold (0.21±0.21mm). No significant differences were found in the mesial, buccal, and palatal surfaces. Conclusions: One Reci proved to be the most conservative system for pericervical dentin, suggesting that system selection can significantly impact dental structure preservation during endodontic treatment.

## RESUMEN

**Introducción:**

El tratamiento endodóntico busca desinfectar y moldear los conductos radiculares mediante el desgaste controlado de sus paredes. La selección del sistema rotatorio adecuado es fundamental para conservar la dentina pericervical.

**Objetivo:**

Comparar el desgaste en dentina pericervical con tres sistemas reciprocantes (WaveOne Gold, One Reci y Reciproc Blue).

**Metodología:**

Estudio experimental *in vitro* con 30 premolares unirradiculares, distribuidos aleatoriamente en tres grupos de 10 dientes cada uno. Se realizaron evaluaciones tomográficas antes y después de la instrumentación, y se midieron las superficies mesial, distal, palatina/lingual y vestibular a 4 mm de la unión cemento-esmalte. Se utilizó la prueba de Kruskal-Wallis para comparar los tres sistemas, con la prueba *post-hoc* de Dunn para comparaciones por pares (nivel de significancia p < 0,05).

**Resultados:**

Se encontró diferencia estadísticamente significativa en el sector distal (p = 0,005), y fue One Reci el sistema que causó menor desgaste (0,05 ± 0,02 mm) comparado con WaveOne Gold (0,21 ± 0,21 mm). No se encontraron diferencias significativas en las caras mesial, vestibular y palatina.

**Conclusiones:**

One Reci demostró ser el sistema más conservador de dentina pericervical, lo que sugiere que la selección del sistema puede impactar significativamente en la preservación de la estructura dental durante el tratamiento endodóntico.

## INTRODUCTION

Endodontics, as defined by the American Association of Endodontists, is the dental specialty that studies the morphology, physiology, and pathology of dental pulp and periradicular tissues ^1^. Effective endodontic treatment requires adequate instrumentation of the root canal system for disinfection and shaping, a procedure where rotary and reciprocating systems have revolutionized clinical practice in recent years ^2^.

Endodontic instrumentation systems are primarily classified into oscillatory (reciprocating) and continuous rotary systems ^3^. Oscillatory systems offer advantages such as reduced risk of cyclic fatigue fracture and potential preservation of dentinal tissue, while rotary systems excel in tissue removal efficiency and speed ^4^^,^^5^.

Abous Rass identified "danger zones" in the root canal walls susceptible to perforation due to over-instrumentation ^6^, while Keesler described the pericervical zone, located approximately 4 to 6 mm below the pulp chamber floor, as a critical area with dentinal thickness of 1.2-1.3 mm ^7^. This region is particularly important for the structural stability of endodontically treated teeth.

Currently, various instrumentation systems have emerged, each with distinctive characteristics. WaveOne Gold is characterized by its flexibility, fatigue resistance, and cutting efficiency ^8^. One Reci stands out for its high efficiency without screw-in effect, thanks to its C.Wire heat treatment and thin diameter that preserves pericervical dentin(8). Reciproc Blue incorporates an innovative heat treatment that optimizes its flexibility, facilitating safe canal preparation ^9^.

The evaluation of dentinal wear can be performed using non-invasive techniques such as computed tomography, which provides high-resolution three-dimensional images ^10^^,^^11^. Various studies, such as those by Zhang ^12^, Katanec^13^, and Miguens^14^, have evaluated the incidence of dentinal microcracks with these systems, although recent scientific evidence remains limited^15^.

Excessive wear of pericervical dentin may compromise the structural integrity of the tooth and lead to long-term complications. Therefore, selecting a system that combines instrumentation efficacy with preservation of dental structure could be fundamental for treatment success.

This study aims to compare the pericervical dentin wear produced by three reciprocating rotary systems (Wave One Gold, One Reci, and Reciproc Blue) through tomographic evaluation, seeking to determine which offers the best balance between efficacy and preservation of dental structure.

## MATERIALS AND METHODS

### Study Design and Population

A randomized in vitro experimental study was conducted with approval from the Ethics Committee of the Universidad de Cartagena (Protocole #2024-05). Thirty single-rooted premolars, extracted for orthodontic reasons from patients aged 18-30 years, with straight root canals and fully formed roots, were selected. Teeth with fractures, calcified canals, previous endodontic treatment, caries, or morphological alterations were excluded ^16^.

### Sample Preparation and Distribution

The teeth were immersed in physiological saline solution to maintain hydration, changing it every two days ^17^, and subsequently disinfected in 2.5% NaOCl for one minute ^18^. The samples were fixed in wax, numbered from 1 to 30, and randomly distributed into three groups of 10 teeth each: Group I (Wave One Gold), Group II (One Reci), and Group III (Reciproc Blue).

### Initial Tomographic Evaluation

An initial cone-beam computed tomography (CBCT) was performed using a Veraviewepocs 3D R100 (J. Morita) device with the following specifications ^19^: slice interval and thickness (1.000 mm), voltage (90.0 kV), amperage (6.0 mA), exposure time (9.4 seconds), capture size (40 x 40 mm), and voxel size (125 microns). Measurements were taken of the mesial, distal, palatal/lingual, and buccal surfaces at 4 mm from the cemento-enamel junction ^20^.

### Instrumentation Procedure

Cameral access was performed using a #801 round diamond bur. The canals were located and permeabilized with a K-file #10 to determine the working length ^21^. Instrumentation was carried out following the specific protocols for each system:

1. Wave One Gold: Instrumentation with PRIMARY file, applying brushing movements outward to eliminate coronal interferences until reaching the full working length ^8^.
2. One Reci: Sequence with C-Pilot file #10, One G file with progressive movements in three waves, and One Reci 25.06 file with progressive brushing movements until reaching the working length ^9^.
3. Reciproc Blue: Instrumentation with R25.08 using pecking movements (maximum 3 mm) alternated with instrument cleaning and irrigation, repeating the process until reaching the working length ^10^.

Sodium hypochlorite was used as an irrigant throughout the procedure.

### Post-instrumentation Tomographic Evaluation

A second CBCT was performed maintaining the same angulations as in the first measurement ^11^, measuring again the mesial, distal, palatal/lingual, and buccal surfaces to determine the degree of wear produced by each system.

### Statistical Analysis

Data were analyzed using SPSS v26. After verifying the non-normal distribution of variables (Kolmogorov-Smirnov and Shapiro-Wilk tests), the Kruskal-Wallis test was applied to compare the three systems, with a significance level of 0.05. Dunn's post-hoc test was used for pairwise comparisons.

## RESULTS

A total of 30 canals were evaluated before and after instrumentation using the three reciprocating rotary systems. Table 1 shows the general characteristics of the evaluated systems.

**Table 1:** General characteristics of the examined systems

SYSTEM	TYPE	TAPER	SIZES	LENGTH	FEATURES
WaveOne Gold	Reciprocating	Small with 7% Primary with 7% Medium with 6% Large with 5%	Small #21 (yellow) Primary #25 (red) Medium #35 (green) Large #45 (white)	21, 25, 31 mm	Increased safety through greater flexibility and cyclic fatigue resistance
Reciproc Blue	Reciprocating	R25 with 8% R40 with 6% R50 with 5%	R25 #25 (red) R40 #40 (black) R50 #50 (yellow)	21, 25, 31 mm	Ability to pre-curve the instrument
One RECI	Reciprocating	One RECI 20.04 One RECI 25.04 One RECI 25.06 One RECI 35.04 One RECI 45.04	One RECI 20 (yellow) One RECI 25 (red) One RECI 35 (green) One RECI 45 (white)	21, 25, 31 mm	High cutting efficiency without screw-in effect due to C.Wire heat treatment and reciprocating movement

Table 2 shows the central tendency measures of the wear produced by each system on the four evaluated surfaces (lingual, mesial, distal, and buccal).

**Table 2:** Central tendency measures of dentinal wear on different surfaces (mm)

SURFACE	SYSTEM	MEAN ± SD	MEDIAN	MAXIMUM	MINIMUM
Lingual	Wave One Gold	0.20 ± 0.24	0.10	0.72	0
	One Reci	0.11 ± 0.12	0.09	0.47	0
	Reciproc Blue	0.058 ± 0.023	0.06	0.08	0
Mesial	Wave One Gold	0.22 ± 0.22	0.11	0.73	0.04
	One Reci	0.09 ± 0.06	0.07	0.24	0.04
	Reciproc Blue	0.09 ± 0.02	0.09	0.13	0.05
Distal	Wave One Gold	0.21 ± 0.21	0.09	0.61	0.06
	One Reci	0.05 ± 0.02	0.05	0.08	0.02
	Reciproc Blue	0.07 ± 0.02	0.07	0.13	0.04
Buccal	Wave One Gold	0.14 ± 0.18	0.06	0.61	0
	One Reci	0.08 ± 0.08	0.06	0.32	0
	Reciproc Blue	0.04 ± 0.02	0.04	0.08	0.02

Normality tests (Kolmogorov-Smirnov and Shapiro-Wilk) indicated that all evaluated variables did not follow a normal distribution (p < 0.05), similar to what was reported by Miguéns et al. ^14^, so the Kruskal-Wallis test was used to determine if there were significant differences among the three systems.

**Table 3:** Statistical results of the comparison between systems

SURFACE	TEST STATISTIC^a^	DEGREES OF FREEDOM	P-VALUE	SYSTEM COMPARISON^b^	ADJUSTED P-VALUE
Mesial	3.012	2	0.222	Not applicable	Not applicable
Distal	10.798	2	0.005*	One Reci vs. Reciproc Blue	0.150
				One Reci vs. Wave One Gold	0.003*
				Reciproc Blue vs. Wave One Gold	0.575
Buccal	1.658	2	0.437	Not applicable	Not applicable
Palatal	3.267	2	0.195	Not applicable	Not applicable

a Kruskal-Wallis test

b Dunn's post-hoc test (only for distal surface)

*Statistically significant (p < 0.05)

The results of the Kruskal-Wallis test revealed a statistically significant difference in pericervical dentin wear only on the distal surface (p = 0.005), while no significant differences were found on the mesial (p = 0.222), buccal (p = 0.437), and palatal (p = 0.195) surfaces, similar to the findings of AlRahabi ^11^.

Pairwise comparison showed a statistically significant difference between the One Reci and Wave One Gold systems on the distal surface (p = 0.003), with significantly less wear with the One Reci system (0.05±0.02mm) compared to Wave One Gold (0.21±0.21mm), coinciding with the results obtained by Koohnavard et al. ^18^.

**Figure 1:**
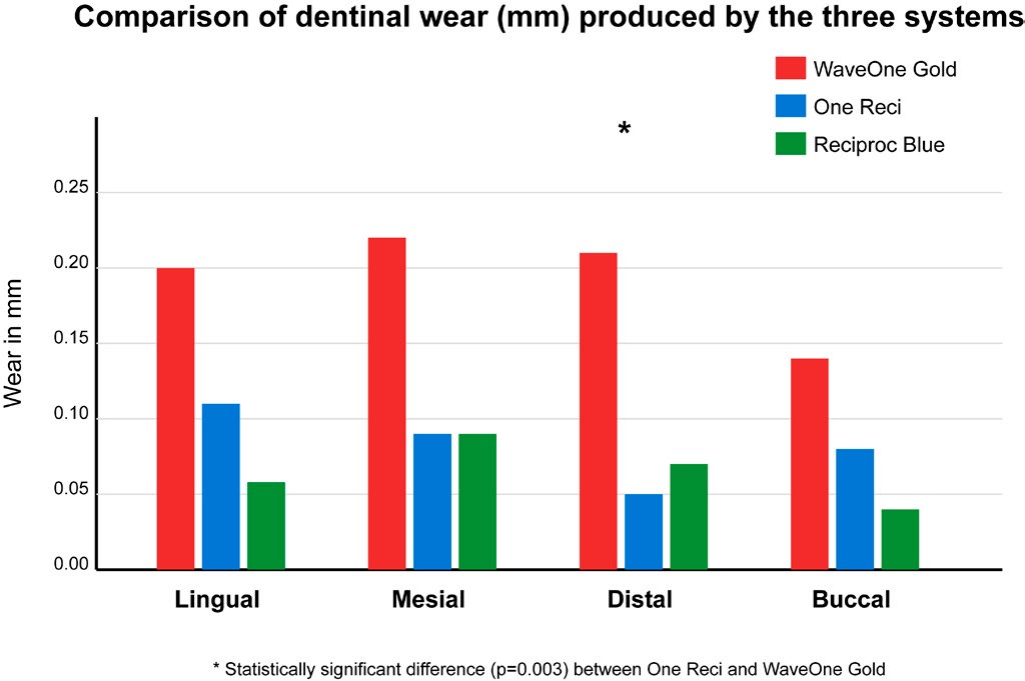
Comparison of dentinal wear (mm) produced by the three systems on different surfaces. The results indicate that One Reci caused the least pericervical dentin wear, particularly on the distal surface (0.05±0.02mm) with a statistically significant difference compared to WaveOne Gold (0.21±0.21mm, p=0.003), suggesting that it better preserves the dental structure during endodontic instrumentation.

## DISCUSSION

This study evaluated the pericervical dentin wear produced by three reciprocating rotary systems: Wave One Gold, One Reci, and Reciproc Blue, in a critical area for the structural integrity of endodontically treated teeth ^6^^,^^7^. The results revealed statistically significant differences in dentinal wear among the systems, particularly on the distal surface, where One Reci showed significantly more conservative behavior than WaveOne Gold.

The Wave One Gold system produced greater average wear on all evaluated surfaces (0.19-0.22 mm), with high variability (SD: 0.18-0.24 mm), coinciding with what was reported by Vorster et al. ^8^, who evaluated the behavior of this system in different types of access. This greater dentin removal could be attributed to its specific design characteristics and the "Gold" thermomechanical process, which, while improving flexibility, appears to be more aggressive with the dentinal structure. Castañeda ^9^ described that this system was primarily designed to optimize cutting efficiency and fatigue resistance, which would explain its more aggressive behavior with dentin.

One Reci proved to be the most conservative system, especially on the distal surface (0.05±0.02 mm), with statistically significant differences compared to Wave One Gold (p=0.003). These findings are consistent with Koohnavard et al. ^18^, who found that systems with similar characteristics better preserve dentin. Ramos Castañeda et al. ^9^ highlight that the C.Wire heat treatment and its particular cutting design allow maintaining efficiency without excessive wear, a particularly valuable characteristic in the pericervical region where structural conservation is crucial.

Reciproc Blue showed intermediate behavior, being particularly conservative on lingual (0.058±0.023 mm) and buccal (0.04±0.02 mm) surfaces. De-Deus et al. ^10^ point out that the "Blue" heat treatment confers greater flexibility, explaining its more controlled behavior during instrumentation. Moradas Estrada (2) indicates that this property is advantageous in curved or narrow canals, where the risk of transportation and excessive wear is greater.

AlRahabi^11^ reported less surface distortion with Reciproc Blue compared to WaveOne Gold, attributing these differences to variations in manufacturing methods and heat treatments. Firstov et al. ^15^ explain that these treatments modify the crystallographic structure of NiTi alloys, altering their mechanical properties and behavior during instrumentation.

The preservation of pericervical dentin is fundamental for maintaining the integrity of endodontically treated teeth, reducing the risk of long-term fractures, as indicated by Lang et al. ^20^. Pérez et al. ^3^, emphasize that the endodontic treatment triad must contemplate not only the effectiveness in cleaning and disinfection but also the maximum preservation of healthy dental structure, an aspect in which One Reci and Reciproc Blue showed significant advantages over WaveOne Gold in our study.

The clinical implications of these results are relevant for daily endodontic practice. According to our findings, the One Reci system might be preferable in cases where pericervical dentin preservation is critical, such as in teeth with compromised structure or thin walls. The tomographic methodology employed allowed precise and objective measurements of the wear produced by each system, coinciding with Espitia Mendoza et al. ^19^, who highlight the advantages of this technology for quantitative evaluations in endodontics.

## STUDY LIMITATIONS

This study presents limitations that should be considered when interpreting results. The in vitro nature limits extrapolation to clinical conditions, lacking pulp tissue and dynamic oral environment factors. The sample size (30 teeth, 10 per group) may limit statistical power for detecting smaller clinically relevant differences. The study was restricted to single-rooted premolars with straight canals, limiting generalizability to molars and curved anatomies. Single operator performance reduces inter-operator variability but may not reflect varied clinical outcomes. Measurement protocol was limited to one level (4 mm from CEJ) and didn't evaluate canal transportation or instrumentation time. Tomographic methodology has resolution limitations for detecting minimal structural changes. Finally, long-term clinical implications regarding fracture resistance and treatment success were not assessed.

## CONCLUSIONS

The One Reci system produced less pericervical dentin wear compared to WaveOne Gold, with statistically significant differences on the distal surface, demonstrating its capacity to better preserve the dental structure during endodontic instrumentation.

The WaveOne Gold system generated greater average wear and greater variability on all evaluated surfaces, suggesting less predictable behavior and potentially more aggressive interaction with pericervical dentin, a critical factor for the structural resistance of the tooth.

The selection of the rotary system should consider not only its effectiveness in cleaning and shaping but also its capacity to conserve pericervical dentin. Further research with larger samples is recommended to confirm these findings.

## Sources of financing:

This research was self-financed by the authors.
